# Association of ACE gene polymorphism with cardiovascular determinants of trained and untrained Iranian men

**DOI:** 10.1186/s41021-019-0126-7

**Published:** 2019-04-04

**Authors:** Akram Falahati, Hamid Arazi

**Affiliations:** 1 Department of Exercise Physiology, University Campus, University of Guilan, Rasht, Iran; 2 Department of Exercise Physiology, Faculty of Sport Sciences, University of Guilan, P.O. Box: 41635-1438, Rasht, Iran

**Keywords:** Genetic polymorphism, Oxygen consumption, Soccer, Trained and untrained, Physical performance

## Abstract

**Background:**

The insertion (I) rather than deletion (D) of human angiotensin converting enzyme gene (ACE) is associated with lower circulating ACE activity and with endurance performance among Caucasians. The frequency of the ACE gene I/D allele in Iranian sample seems to be more similar to the Caucasians. To assess the possible relationship between I/D polymorphism of ACE gene with athletic status and selected cardiovascular indices and VO_2max_ in an Iranian population, DNA samples were obtained from 57 trained and untrained men, with soccer as their main training modality. Genotyping for ACE I/D polymorphism was performed using polymerase chain reaction. VO_2max_ was determined by an incremental test to volitional exhaustion on a motorized treadmill.

**Results:**

I/D genotype was neither associated with elite athlete status nor with VO_2max_, resting heart rate, systolic and diastolic blood pressure. There was no interaction effect of training statue x ACE genotype for each of the examined indices.

**Conclusions:**

ACE gene variation was not a determinant of cardiovascular function and VO_2max_ in either trained or untrained Iranian participating in soccer. The absence of an association between either I/D genotype and elite Iranian athlete status and better cardiovascular function also suggests that the ACE gene does not contribute significantly to the phenomenal success of Iranian soccer players.

## Introduction

Vigorous regular physical exercise is related with physiological cardiovascular adaptations that lead to heart modification which improve physical performance. Progressing endurance, having a lower resting heart rate and a stronger heart volume are all valid physiological indicators of the cardiovascular system’s ability to adjust to physical overload in well-trained athletes [1]; indeed, the type of exercise performed is a great determinant of cardiovascular response to exercise [2]. Soccer is a high-intensity endurance sport that needs intermittent and random bouts of powerful anaerobic activities [3]. When the duration of the game and the significance of expeditious recovery from anaerobic bouts of activity are considered, VO_2max_ is assumed as a routine measure to estimate soccer performance.

The individual variability of physical performance characteristics and the potential to become an elite athlete have a powerful genetic base. The genetic effect of VO_2max_ is about 50% [4]; but, the role of genetics in the achievement of world class status and high athletic performance is not established yet [5]. Angiotensin-converting enzyme (ACE) gene which is the candidate gene involved in cardiac growth and function, has been broadly studied for cardiorespiratory fitness phenotypes. The endocrine renin angiotensin system (RAS) plays an essential role in controlling the circulatory system. Genes encoding components of RAS regulate ACE levels. In intron 16 of the ACE gene, a polymorphism consisting in the presence (insertion) or absence (deletion) of the 287-bp Alu sequence has been detected. The D allele is associated with peak ACE levels in ventricular tissue as well as in the circulation [6]. The I allele has been linked to an enhanced endothelium-dependent vasodilation [7], high percentage of type I muscle fibers [8], therefore, suggesting that type II carriers would present higher VO_2max_.

While ACE is involved in the metabolism of substances that impact on vascular remodeling, it may regard in the cardiorespiratory fitness of individuals and for the differences among individuals in response to physical training [9]. High frequency of the ACE I allele has been reported in mountaineers [10, 11] and rowers [12], in comparison to non-athlete controls. The moderately active young men with DD genotype had a significantly lower VO_2max_ [13]. In contrast, a higher VO_2max_ for the DD genotype (57 ml.kg^-1.^min^− 1^) versus the ID or II (50 ml.kg^− 1^ min^− 1^) genotype was demonstrated in Chinese males [9]. Other studies did not find any relation between ACE I allele and VO_2max_ [5, 14] or elite athlete status [15].

Besides, cardiac response to certain physiological stimuli is affected by racial differences. It seems that certain ethnicities demonstrate a stronger association between ACE activity or I/D polymorphism and athletic results [16]. Saadat et al. (2015), reported that the frequency of the D allele in Iranian sample (about 62.6%) seems to be more similar to the Caucasians (40–60%) than the Asians (25–40%) [17]. In a study on Iranian elite athletes in three groups of mixed (basketball, volleyball and taekwondo), endurance and power or sprint performance, it was demonstrated that I allele in athletes is significantly more than controls. Frequency of the I allele (53.13%) was higher than the D allele in mixed-oriented athletes. Distribution of the I and D alleles in power-oriented athletes was equal (50%). While in endurance-oriented athletes, there was a clear increase in proportion of the D allele (63.51%) [18].

In a population association study, a homogeneous cohort of subjects from the same sporting discipline is needed when investigating whether a genetic marker (the ACE I/D polymorphism) occurs more frequently in cases (elite athletes) than in controls [19]. Therefore, it is necessary to use homogeneous samples and protocols to reduce any differences resulting from the phenotypic characteristics of participants. Thus, the aim of the present study was to examine the possible influence of ACE genotype (I/D) on cardiovascular determinants of trained and untrained Iranian men.

## Methods

### Participants

Soccer players (white men of Iranian descent) were recruited through an open invitation from a large soccer club. In total, 57 subjects gave written informed consent to participate after the study had been explained in details. Physical activity levels, medical health and quality of life of the subjects was assessed by a validated questionnaire (36-Item Short-Form Health Survey questionnaire [20]. The ACE-I/D polymorphism was determined in a double-blind manner from blood and after the VO_2max_ and other cardiovascular determinants had been measured was assessed post-hoc for its influence.

For the analysis, subjects were categorized based on their training status and ACE I/D genotype as assessed by questionnaire, functional exploration and genotyping. Volunteers included highly trained male (*n* = 29) soccer players, competitors in the first national league who had at least a history of 5 years of regular training (with soccer as their main training modality), recorded a level of intense physical activity above 8 h per week (in the questionnaire). Untrained subjects (*n* = 28) were active members of the community (soccer) who were not involved in a regular training program. In this study, the untrained group with a level of intense physical activity below 2 h per week and a range of VO_2max_ of (29.2–49.7) is in consistence with the study of Valdivieso (2017) who considered healthy subjects which documented a level of intense physical activity below 6 h per week or demonstrated a VO_2max_ below 50 ml·Kg^− 1^·min^− 1^ as not being trained, i.e., being untrained [21].

Untrained subjects did not differ from the trained group in their ethnic and geographical distribution throughout Iran. All subjects were free from any musculoskeletal, metabolic, and cardiorespiratory disorders. All participants were asked to abstain from strenuous exercise as well as alcohol and caffeine intake for at least 48 h prior to the test session. The study was approved by the Ethical Committee at the University of Guilan.

### Study design

#### Anthropometric and physical performance measurement

Height (stretched stature), mass and skinfold measurements were taken according to standard procedures used by ISAK [22]. Skinfold thickness at four sites (biceps, triceps, subscapular and suprailiac) were marked and measured by a trained anthropometrist. Duplicate measurements were obtained from each site and a third measure was conducted if the technical error of measurement advised by ISAK was exceeded. Skinfold caliper (Slim guide, C-120; Michigan, USA) was used in collection of data. The percentage of body fat was calculated from these measurements using the equation of Durnin and Womersley [23]. Following instructions by the researcher, self-determination of resting heart rate (RHR) was conducted by subjects, for three mornings before getting up from bed. Blood pressure was measured on the right arm in the sitting position after 5 min rest before the exercise protocol using an automated brachial oscillometric device (M10-IT; Omron, Kyoto, Japan). Systolic blood pressure (SBP) and diastolic blood pressure (DBP) were calculated from two recordings with a minimal interval of 10 min.

Aerobic fitness level of each soccer player was determined by measuring VO_2max_ during a graded incremental exercise test on a treadmill (HP Cosmos, Germany), using Bruce protocol in line with the ACSM guidelines [24]. The testing session occurred during a preparatory period, 2–3 h after a standardized breakfast, in an air-conditioned laboratory with the temperature and relative humidity set at 20 °C and 50%, respectively. The test consists of 7 stages, each lasting for 3 min and the speed of the track is significantly increased with each subsequent stage, forcing the subject to speed up his steps. The exercise is stopped when the subject accomplished the following criteria: an increase in VO_2_ of less than 2 ml · Kg^− 1^ · min^− 1^ with increasing running speed (VO_2_ plateau); a final heart rate (bpm) defined as a heart rate within 10 beats.min^− 1^ of the sex and age predicted maximum heart rate (HR_max_ = 220- age). Heart rate was measured and recorded by a HR monitor (Polar S610i, Kempele, Finland). The final result of the Bruce test is exercise time (ET) which is necessary for determining the value of maximal oxygen consumption (VO_2max_).

### Genotyping of the ACE gene I/D polymorphism

Venous blood from all participants in this study, obtained by venipuncture of the antecubital vein in a sitting position were collected in EDTA tubes and stored at − 20 °C. Genomic DNA was extracted from whole blood leukocytes using the salting-out method as previously described by Miller et al. [25]. We used Polymerase Chain Reaction (PCR) to genotype DNA samples, and clarify the presence of the D (190 bp) and I (490 bp) allele. Polymorphism was determined using a primer pair described by McCauley [21]. The PCR reaction conditions are shown in Table 1. The PCR product was analyzed by electrophoresis at 80 V for 60 min using 1.5% agarose gel. Blind samples were used to control the reliability of genotyping. This research was performed in the molecular laboratory of Guilan University, Iran.

**Table 1:** The polymerase Chain Reaction (PCR) conditions

Method	Forward Primer (5′-3′)	Reverse Primer (5′-3′)	PCR reaction conditions		
McCauley et al. [25]	CTGGAGACCACTCCCATCCTTTCT	GATGTGGCCATCACATTCGTCAGAT	Denaturation 95 °C for 1 min	Annealing 30 cycles of	Extension 72 °C for 10 min
				95 °C for 1 min	
				58 °C for 1 min	
				72 °C for 2 min	

### Statistical analysis

Data are presented as mean ± standard deviation (SD). The significance of differences in allele frequencies was estimated using the χ2 test. Two-way analysis of variance (factors: training state and ACE I/D polymorphism) was used to assess mean differences. Subjects were tested at both loci for Hardy Weinberg Equilibrium (HWE) by Chi-square tests with one degree of freedom. Statistical significance was set at *P* < 0.05. Data were analyzed using SPSS version 16.0 (Chicago, IL, USA).

## Results

The frequency distribution of ACE genotypes in the 57 Iranian males in the present study was: DD (31%, *n* = 18), ID (46%, *n* = 26), II (23%, *n* = 13). No differences were found in the ACE genotype frequencies between trained and untrained males. Also, the allele frequency was statistically similar (Table 2). The distribution of ACE genotype was in Hardy-Weinberg equilibrium.

**Table 2:** Frequency of ACE insertion (I) and deletion (D) alleles and genotypes in trained and untrained males

	Trained (n = 29)			Untrained (n = 28)			All (*n* = 57)			Allele (n = 57)	
	ID	II	DD	ID	II	DD	ID	II	DD	I	D
Participants	13	7	9	13	6	9	26^a^	13	18	39	49
	(0.45)	(0.24)	(0.31)	(0.47)	(0.21)	(0.32)	(0.46)	(0.23)	(0.31)	(0.47)	(0.53)

Note: II, ID, DD – ACE genotype; fractions in parentheses; ^a^ genotype frequency significantly different from ACE II; *p* = 0.037

The trained and untrained men had similar age, height, weight, BMI and resting SBP and DBP. However body fat mass (%), ET and RHR were significantly (*P* < 0.001) higher in untrained and VO_2max_ in trained group (Table 3).

**Table 3:** Characteristics of trained and untrained males

Variable	Trained (n = 29)	Untrained (*n* = 28)	Statistical significance
Age (years)	38.5 ± 16.5	38.9 ± 16.8	**ns**
Weight (kg)	74.9 ± 5.1	76.3 ± 8.9	**ns**
Height (cm)	179.3 ± 3.5	181.3 ± 6.2	**ns**
BMI (kg · m^−2^)	23.3 ± 1.5	23.2 ± 2.0	**ns**
Fat mass (%)	13.4 ± 2.3	18.6 ± 2.8	***p*** < 0.001 *
Exercise time (min)	15.2 ± 1.7	10.9 ± 1.4	***p*** < 0.001 *
VO_2max_ (ml · Kg^−1^ · min^− 1^)	56.0 ± 6.9	39.0 ± 5.8	***p*** < 0.001 *
Resting SBP (mmHg)	126 ± 7	127 ± 6	**ns**
Resting DBP (mmHg)	76 ± 4	77 ± 4	**ns**
RHR (b.min ^− 1^)	57 ± 3	65 ± 3	***p*** < 0.001 *

Values are Means ± SD; * = *P* < 0.05; *BMI* Body Mass Index, *SBP* Systolic Blood Pressure, *DBP* Diastolic Blood Pressure, *RHR* Resting Heart Rate; ns: not significant

The median of VO_2max_ is 49.6 ml.kg^− 1^.min^− 1^ (range 34.2–68.1 ml.kg^− 1^.min^− 1^) in DD genotype group, 46.2 ml.kg^− 1^.min^− 1^ (range 29.2–66.2 ml.kg^− 1^.min^− 1^) in ID group and 48.7 ml.kg^− 1^.min^− 1^ (range 31.5–61.8 ml.kg^− 1^.min^− 1^) in II group.

Age, height, body weight and BMI in our study population, did not differ significantly among the three ACE genotype groups. The ACE genotype had no significant effect on VO_2max_, resting SBP and DBP, fat mass and RHR (Table 4). Those with II genotype tended to have a lower RHR than those with the ID (*P* < 0.09) and DD (*P* < 0.08) genotypes (Table 4).

**Table 4:** Cardiovascular determinants of different ACE genotypes in trained and untrained males

Variables	Trained			Untrained			Effects		
	II	DD	ID	II	ID	DD	Training	Genotype	Interaction
VO2max (ml.kg^−1^.min^−1^)	54.5 ± 5.1	56.4 ± 6.8	57.9 ± 7.5	42.1 ± 6.5	36.1 ± 4.5	41.3 ± 5.6	*p* < 0.001	ns	ns
Resting SBP (mmHg)	130 ± 4	126 ± 7	125 ± 7	124 ± 7	128 ± 5	126 ± 6	ns	ns	ns
Resting DBP (mmHg)	76 ± 4	77 ± 3	75 ± 5	75 ± 3	77 ± 4	78 ± 4	ns	ns	ns
Fat mass (%)	14.1 ± 3.1	12.8 ± 2.1	13.6 ± 2.1	18.5 ± 3.6	18.1 ± 2.9	19.3 ± 2.3	*p* < 0.001	ns	ns
Resting HR (b.min ^− 1^)	56 ± 3	57 ± 2	57 ± 3	62 ± 2	65 ± 2	65 ± 3	*p* < 0.001	ns	ns

Note: II, ID, DD: ACE genotypes; VO_2max_: maximal oxygen uptake; SBP: systolic blood pressure; DBP: diastolic blood pressure; HR: heart rate; ns: not significant

The mean VO_2max_ was significantly higher in trained than in untrained subjects. No interaction effect of training statue x ACE genotype was found for each of the examined indices (Table 4).

## Discussion

The distributions of three ACE genotypes for DD, ID, and II in our study were 31, 45 and 24% versus 32, 47 and 21%, for trained and untrained subjects respectively. The prevalence of the D allele in our sample (53%) is in agreement with previous studies from Iran [17, 26]. Saadat et al. (2015), reported that the frequency of the D allele in Iranian sample (about 62.6%) seems to be more similar to the Caucasians (40–60%) than the Asians (25–40%) [17]. Despite this similarity to Caucasians and their potent association with ACE activity [27], the results of the present study do not support an association between elite athlete status and variations in the ACE gene among Iranians which is in contrast with some studies [10–12, 18, 28] who have reported differences in the allele frequency of performance genes such as ACE when excellent athletes were compared to controls. An increased frequency of the ACE I allele has been observed in high altitude mountain climbers [10, 11] and rowers [12], in comparison to non-athlete controls.

Shahmoradi et al. (2014) grouped Iranian elite athletes in three categories of mixed, endurance and power sports. It was demonstrated that frequency of the I allele (53.13%) was higher than the D allele in mixed-oriented athletes. Besides, I allele in athletes was significantly higher than controls [18]. According to this categorization, soccer is classified as mixed group. But contrary to this study the frequency of the D allele in our sample (53%) was higher than I allele (47%). It may be because of the heterogeneous sample in the study of Shahmoradi because, a population association study testing whether a genetic marker (the ACE I/D polymorphism) occurs more frequently in cases (elite athletes) than in controls, requires a homogeneous cohort of subjects from the same sporting discipline [19].

Cardiovascular performance plays an important role in athletic success [29]. Bradykinin, as a vasodilator, improves peripheral circulation and thus enhances oxygen and substrate delivery to the working muscles [14]. In spite of the role of RAS in modifying metabolic function of heart during exercise, this system has not been well characterized in relation to cardiovascular determinants and VO_2max_ which is considered the gold standard for cardiorespiratory evaluation. Improvements in VO_2max_ have been associated with improved soccer performance during competition (i.e., distance covered, average work intensity, involvement with the ball) [30]. Physical performance results from an interrelationship between genetic factors and environmental stimuli. This complicated interaction create a distinction between individuals and populations [26].

In our study, we did not find any association between the ACE gene I/D polymorphism and cardiovascular determinants in univariate analysis. These results are in accordance with the results from other studies, where no association was found between ACE gene variation and endurance performance [14, 31]. It is unclear what mechanisms were underlying the lack of association between ACE I/D polymorphism and cardiovascular determinants in samples. A non-homogeneous sample in terms of training status is one. On the other side this might be due to the homogeneity of the sample based on the sport modality. But Woods et al. (2001) confirmed the necessity of using homogeneous samples and protocols to minimize any differences resulting from the phenotypic characteristics of participants [19]. As a result, instead of selecting athletes from a variety of sports, we employed soccer players to be included in the present study. The “untrained” subjects are also member of soccer club but the physical activity is not much greater than the general populations including the populations having stationary life style. Several recent studies [32, 33] has made comparisons between elite soccer players and their non-elite counterparts and have named them as amateur or sub-elite soccer players. Ostojic (2003) indicated that elite soccer players were older than their amateur counterparts. In other words, amateur leagues are place for young talented players to improve their knowledge and skills. Both sub-elite and elite soccer players compete at the national elite level in their respective age. But our trained (38.5 ± 16.5 years) and untrained (38.9 ± 16.8) group were of the same age.

Wells et al. (2012) [34] indicated that professional and amateur soccer players did not differ in VO_2max_ (professional 56.5 ± 2.9 ml · Kg^− 1^ · min^− 1^; amateur 55.7 ± 3.5 ml · Kg^− 1^ · min^− 1^: *P* = 0.484). Our trained group with a VO_2max_ of (56.0 ± 6.9 ml · Kg^− 1^ · min^− 1^) supports the above statement. But the untrained group with a VO_2max_ of (39.0 ± 5.8 ml · Kg^− 1^ · min^− 1^) is far from the above mentioned data and is closer to the healthy untrained men at this age [35].

Perhaps it would have been better if we used sports that their athletes had higher levels of VO_2max_. But even in a study by Rankinen et al. (2000) on 192 elite endurance athletes with VO_2max_ values over 83 ml.kg^− 1^.min^− 1^ (highest decile), they did not find any association between ACE genotype and VO_2max_ [14]. These findings argue against the idea that the ACE I/D polymorphism is associated with extraordinary cardiorespiratory endurance performance.

In addition to the ACE variants, many other genetic variants may be responsible for interindividual variability of VO_2max_ levels. Williams et al. (2017) [36] conducted a systematic review which aimed to summarize genetic variants that have been identified as influencing VO_2max_. They reviewed 35 studies that reported 97 genes associated with improvements in VO_2max_. It has been estimated that VO_2max_ trainability has a significant heritable component of around 50% [37]. It is confirmed that the response to exercise is so complex and polygenic (i.e. influenced by many genes working together) and each genetic variant has a small impact (typically less than 1%) to the overall change in VO_2max_.

Despite inconsistency among past reports, an association between the ACE I/D polymorphism and circulating ACE levels has been almost accepted. The I allele has been associated with endurance phenotypes and the D allele with power. A greater maximal oxygen extraction (a-vO2 difference) in ACE II versus ID and DD genotypes was reported [38]. Also, many previous studies have supported a significant association for increased endurance-based physical performance with the ACE II genotype compared to D-allele carriers (DD + ID) [12, 13, 28]. Therefore, it may have been expected that the I allele would be associated with higher VO_2max._

On the other hand, there are some other studies that contradict these findings and suggest an association of D allele/DD genotype with endurance athletes’ phenotypes [9, 19, 39]. However, there have been some studies that suggest no association between the ACE I/D polymorphism and physical performance [5, 14, 15, 31].

Traits such as response to exercise do not only depend on the genetic code, but also on epigenetic signals. Epigenetic modulation of gene activity occurs in response to non-genetic factors such as physical activity [40], body weight, diet, and environmental toxins [41]. Both acute and chronic exercises significantly impact DNA methylation. Besides, the response to exercise can also be affected by epigenetic factors. It means that in addition to nutrition and physical activity status, there are other differences that should be taken into consideration including: age, training duration and volume (MICT vs. HIIT), weight, fat percentage, medications, clinical versus healthy populations; sleep and psychological status. Together, these are potential epigenetic modifiers (e.g. DNA methylation and histone acetylation) that can influence gene expression, molecular function and thereby influence VO_2max_ response.

Ethnic as well as geographic differences might be responsible for the observed disagreements [42]. The evidence about racial differences in cardiac response to certain physiological and pathological stimuli [16] may help explain some of the controversy about the association between ACE genotype and physical performance. Certain ethnicities appear to demonstrate a stronger association between ACE activity or I/D polymorphism and athletic results [16]. The link between ACE I/D genotype and ACE activity is not strong enough in African subjects [31], which is the reason for no association response between ACE I/D genotype and performance in those populations. Many of the studies supporting the hypothesis by which I/D allele would be associated with better cardiovascular response to exercise, have been conducted in Caucasian subjects where the ACE genotypes are strongly associated with ACE activity [27]. However, generalizing an association found in one population to others must be treated with caution. For the gene studies, it is more critical to avoid false-positives. The presence or absence of an observed association in any ethnic, racial or geographic population may be related to other genes and environmental factors [42], low power due to small sample sizes, heterogeneity between the samples or differences in ethnic composition influencing the amount of linkage disequilibrium (LD) between the markers studied and the DNA changes contributing to the phenotype.

Gene-specific patterns of LD make it difficult to provide general guidelines for selecting the most useful polymorphisms in association studies. So, these kinds of studies should concentrate on the overall sequence variation of functionally important regions of candidate genes and not only on a few polymorphisms. Evaluating the pattern of LD in the human genome is important to identify tagging SNPs. these SNPs are often correlated due to linkage disequilibrium (LD). But in the present study the LD structure among SNPs was not examined.

Many physical and physiological traits have a multifactorial etiology resulting from the interplay of genetic factors (G) and environmental exposures (E). Differential classification may increase the power to detect true interactions in a genetic based study. An implication of our null finding is that, in real-life situations that is in studies by which more genetic markers will have detectable non-null effects for a given sample size, the number of markers identified will be good deal more than those obtained from G-E interaction methods. One must then investigate which markers are implicated in G-E interaction.

Several limitations of this study exist. We did not consider tests for additive interaction. There are many possible reasons why gene studies lack power to detect G-E interactions, including small sample size or more complex multimarker interactions.

Using convenient single-nucleotide polymorphism simulation routines that generate true LD structure would make our study more realistic, changing it from a fixed null scenario to a reasonable genetic interaction.

In addition to the endurance performance traits, the data on the effects of the ACE I/D polymorphism on various cardiovascular phenotypes in general are incoherent [14]. Previously, in the Iranian population, it has been shown that the D allele is associated with cardiovascular dysfunction [43], and left ventricular hypertrophy (LVH) [44]. Moreover, the reports about Iranian subjects showing the association of II genotype with severity of vein graft atherosclerosis [45], the independent association of DD polymorphism with hypertension in the diabetic population [42], further add to the confusion. All of which suggest the role of genetic factors in cardiac function. Thus it is obvious that more studies are needed to completely comprehend the function of the ACE gene and the possible effects of its DNA sequence variation on various physical and physiological traits.

### Limitations

This investigation was performed under several constraints. Lack of serum ACE activity measurement and also the small sample size are main limitations of this study. When small sample size is used, the risk is high that observations will be due to chance and the estimates derived may be biased. So the data should be interpreted cautiously due to the small sample size. Therefore, there is an urgent need for more investigations on markedly larger sample size to understand the function of the ACE gene and its possible effects on cardiac function and athletic performance.

## Conclusion

In conclusion, although there are conflicting results about the effects of ACE I/D polymorphism on cardiovascular variables, the current study does not support the hypothesis that ACE I/D genotype is either a determinant of cardiovascular function or aerobic performance (VO_2max_) in Iranian men. The absence of an association between either I/D genotype and elite Iranian athlete status also suggests that the ACE gene does not contribute significantly to the phenomenal success of Iranian soccer players. Nevertheless, demographic and ethnic profile should be taken into account in large samples to confirm these results.
